# The immunoglobulin M-degrading enzyme of *Streptococcus suis*, Ide_*Ssuis*_*,* is involved in complement evasion

**DOI:** 10.1186/s13567-015-0171-6

**Published:** 2015-04-19

**Authors:** Jana Seele, Andreas Beineke, Lena-Maria Hillermann, Beate Jaschok-Kentner, Ulrich von Pawel-Rammingen, Peter Valentin-Weigand, Christoph Georg Baums

**Affiliations:** Institute for Microbiology, Centre for Infection Medicine, University of Veterinary Medicine Hannover, 30173 Hannover, Germany; Department of Pathology, University of Veterinary Medicine Hannover, 30559 Hannover, Germany; Department of Structure and Function of Proteins, Helmholtz Centre for Infection Research, 38124 Braunschweig, Germany; Department of Molecular Biology and Umeå Centre for Microbial Research, Umeå University, 90187 Umeå, Sweden; Institute for Bacteriology and Mycology, Centre of Infectious Diseases, College of Veterinary Medicine, University Leipzig, An den Tierkliniken 29, 04103 Leipzig, Germany

## Abstract

*Streptococcus* (*S.*) *suis* is one of the most important pathogens in pigs causing meningitis, arthritis, endocarditis and serositis. Furthermore, it is also an emerging zoonotic agent. In our previous work we identified a highly specific IgM protease in *S. suis*, designated Ide_*Ssuis*_. The objective of this study was to characterize the function of Ide_*Ssuis*_ in the host-pathogen interaction. Edman-sequencing revealed that Ide_*Ssuis*_ cleaves the heavy chain of the IgM molecule between constant domain 2 and 3. As the C1q binding motif is located in the C3 domain, we hypothesized that Ide_*Ssuis*_ is involved in complement evasion. Complement-mediated hemolysis induced by porcine hyperimmune sera containing erythrocyte-specific IgM was abrogated by treatment of these sera with recombinant Ide_*Ssuis*_. Furthermore, expression of Ide_*Ssuis*_ reduced IgM-triggered complement deposition on the bacterial surface. An infection experiment of prime-vaccinated growing piglets suggested attenuation in the virulence of the mutant 10Δ*ide*_*Ssuis*_. Bactericidal assays confirmed a positive effect of Ide_*Ssuis*_ expression on bacterial survival in porcine blood in the presence of high titers of specific IgM. In conclusion, this study demonstrates that Ide_*Ssuis*_ is a novel complement evasion factor, which is important for bacterial survival in porcine blood during the early adaptive (IgM-dominated) immune response.

**Electronic supplementary material:**

The online version of this article (doi:10.1186/s13567-015-0171-6) contains supplementary material, which is available to authorized users.

## Introduction

*Streptococcus* (*S*.) *suis* colonizes different mucosa of pigs, its main host. Virulent strains might, however, cross the mucosal barrier, cause bacteremia and infect various tissues leading to severe pathologies, such as meningitis, arthritis, endocarditis and serositis. Suppurative meningitis caused by *S. suis* is one of the most important diseases in modern swine production as it is associated with severe economic losses. *S. suis* exhibits a high degree of diversity among and within different serotypes. Serotype 2 is worldwide the most important serotype isolated from affected tissues in piglets and also an important zoonotic agent [1-3].

Numerous proteins involved in interaction with the host have been functionally characterized [4,5]. Recently, we identified a 124 kDa large Immunoglobulin M-degrading enzyme of *S.* suis, designated Ide_*Ssuis*_ [6]. The N-terminal region of Ide_*Ssuis*_ is homologous to the 38 kDa IgG specific endoprotease IdeS (also known as Mac-1) expressed by *S. pyogenes* and sufficient for IgM cleavage. Ide_*Ssuis*_ is a highly specific IgM protease expressed by all investigated *S. suis* strains, which included strains from four different serotypes and clonal complexes. Importantly, it is so far the only known protease cleaving specifically the intact IgM multimer. The specificity of this protease is underscored by several findings: (i) Ide_*Ssuis*_ does not degrade porcine or human IgG or IgA, (ii) IgM of pigs but not IgM of any other investigated species is cleaved and (iii) incubation of different body liquids with Ide_*Ssuis*_, including cerebrospinal and joint fluids from diseased piglets, generated only one additional band in SDS-PAGE in accordance with a specific IgM cleavage product [6].

Complement activation leads to formation of C3 convertases (C3Bb or C4b2a) cleaving C3 into the anaphylatoxin C3a and the most important opsonin C3b. In an experimental mouse model the complement system proved to be crucial for protection against morbidity and mortality caused by intranasal *S. suis* infection, as recently demonstrated by our group using C3^−/−^ mice [7]. Thus, evasion of complement activation is essential for the survival of *S. suis* in its host and several factors involved in complement evasion have been identified in *S. suis*. Sialic acid moieties of the capsule of serotype 2 strains [8] might interfere with the activation of the alternative complement cascade by increasing the affinity constant of C3b to the complement inhibitor factor H [9,10]. Accordingly, deposition of C3b is increased on the bacterial surface of an unencapsulated mutant [7]. Furthermore, two factor H binding proteins (Fhb and SSU0186) both homologous to PspC (Pneumococcal surface protein C) of *S. pneumoniae* have been identified in *S. suis* [11,12]. FhB was shown to contribute to virulence in experimental infections of piglets and to survival in human blood ex vivo.

The classical complement pathway is activated by immunoglobulins, in particular IgM, and some other host proteins, e. g. choline-binding protein, recognizing bacterial surface structures [13]. Binding of the IgM pentamer to surfaces of pathogens leads to activation of the classical complement (c) cascade, as IgM, including porcine IgM, contains a C1q binding motif [14,15]. The results of this study showed that the cleavage site of Ide_*Ssuis*_ in porcine IgM is located between the C1q-binding motif and the antigen recognizing part. Thus, we investigated whether IgM protease activity represents a novel complement evasion mechanism protecting the pathogen against classical complement activation.

## Materials and methods

### Bacterial strains and growth conditions

*S. suis* strain 10 is a virulent serotype 2 strain that has previously been used for experimental infections of piglets and for generation of isogenic mutants [16-19]. It expresses the virulence-associated muramidase-released protein (MRP), the extracellular factor and suilysin [20]. The capsule deficient isogenic mutant 10*cps*Δ*EF* is attenuated in virulence [19] and shows increased deposition of C3 antigen on its bacterial surface in murine serum [7]. Streptococci were grown on Columbia blood agar plates or in Bacto^TM^ Todd Hewitt broth (THB). *Escherichia* (*E.*) *coli* strains were cultured in Luria-Bertani (LB) medium. If appropriate, antibiotics were added at the following concentrations: ampicillin, 100 μg/mL for *E. coli*; chloramphenicol, 3.5 μg/mL for *S. suis*, 8 μg/mL for *E. coli*; spectinomycin 100 μg/mL for *S. suis.*

### DNA techniques and primer

Standard DNA manipulations were performed as described [21]. Oligonucleotide primers were designed based on the sequence of SSU0496 in the genome of *S. suis* P1/7 [6]. Chromosomal DNA of strain 10 served as template in all PCRs conducted for generation of inserts. DNA fragments were amplified with Phusion polymerase (Promega, Mannheim, Germany).

### Generation of *S. suis* mutants expressing truncated Ide_*Ssuis*_

The mutant 10Δ*ide*_*Ssuis*_ and its complemented strain 10Δ*ide*_*Ssuis*_ pGA14*ideSsuis* were described previously [6]. In frame deletion mutants expressing either the N-terminal part homologous to IdeS (10Δ*ide*_*Ssuis*__C-terminus) or the large C-terminal part (10Δ*ide*_*Ssuis*__homologue) were generated within this work using the thermosensitive plasmids pSET5Δ*ide*_*Ssuis*__C and pSET5Δ*ide*_*Ssuis*__h, respectively, to mutagenize *S. suis* strain 10. The following amplicons were generated with the indicated oligonucleotide primers to generate pSET5Δ*ide*_*Ssuis*__C: a 619 bp ide_*Ssuis*_ 5′-fragment amplified with ide_*Ssuis*_delCforPstI and ide_*Ssuis*_delCrevBamHI and a 608 bp ide_*Ssuis*_ 3′-fragment generated with ide_*Ssuis*_delCforBamHI and ide_*Ssuis*_delCrevEcoRI (Additional file 1). Both fragments were cut with the restriction enzymes indicated in the name of the primers and inserted into the corresponding sites of pSET5. For the construction of pSET5Δ*ide*_*Ssuis*__h a 614 bp 5′-ide_*Ssuis*_ amplification product was generated with the primer pair preProIde_*Ssuis*_PstI plus postSSide_*Ssuis*_BamHI and a 621 bp 3′-ide_*Ssuis*_ amplification product with the primer pair Ide_*Ssuis*_delh_for_BamHI and Ide_*Ssuis*_delh_rev_SacI (Additional file 1). Both fragments were cut with the indicated restriction enzymes and inserted into the corresponding sites in vector pSET5. Restriction analysis and sequencing was performed with pSET5Δ*ide*_*Ssuis*__h and pSET5Δ*ide*_*Ssuis*__C to verify both constructs.

The allelic exchanges for generation of 10Δ*ide*_*Ssuis*__homologue and 10Δi*de*_*Ssuis*__C-terminus were performed essentially as described previously [6]. The deletion of the genes was confirmed by PCR and Southern Blot analysis, which included four different probes for each mutant strain.

### Generation of an unencapsulated *ide*_*Ssuis*_ mutant

In frame deletion mutagenesis of *ide*_*Ssuis*_ was conducted in the unencapsulated *S. suis* strain 10*cps*Δ*EF* with the thermosensitive plasmid pSET5Δ*ide*_*Ssuis*_ constructed in our previous study [6]. The unencapsulated double mutant 10*cps*Δ*EF*Δ*ide*_*Ssuis*_ was confirmed by comprehensive Southern blot analysis using 4 different probes and two different digestions of DNA (HincII and BamHI).

### Expression and purification of recombinant proteins

The expression and the purification of the different recombinant Ide_*Ssuis*_ constructs, MRP and the fibronectin-and fibrinogen-binding protein of *S. suis* (FBPS) were performed as previously described [6].

### Sodium dodecyl sulphate polyacrylamide gel electrophoresis (SDS-PAGE) and Western blot analysis

For αIgM Western blot analysis samples were prepared with reducing or non-reducing sample buffer and separated in 6% or 10% separating gels. For the detection of the Ig light chain the samples were prepared with reducing sample buffer and separated in a 12% separating gel. Western blot analysis was conducted as previously described [6] with antibodies specified together with the final dilution in Additional file 2. Polyclonal antisera were raised against Ide_*Ssuis*_, Ide_*Ssuis*__homologue and Ide_*Ssuis*__C-terminus in rabbits within our previous study [6].

### Determination of the IgM-cleavage site

The cleavage site of Ide_*Ssuis*_ in IgM was determined through N-terminal sequencing after Edman degradation of a cleavage product. For this, recombinant Ide_*Ssuis*_ in a concentration of 0.07 mg/mL was incubated with 0.68 mg/mL purified porcine IgM at 37 °C for 3 h on a rotator. The proteins were then separated under reducing conditions in a 10% separating and 4% stacking gel. The cleavage products were transferred to PVDF-membranes (Merck Millipore, Schwalbach, Germany) and either visualized in an αIgM Western blot or cut out for sequencing via N-terminal Edman degradation performed on an Applied Biosystems Procise Protein Sequencer 494C with reagents supplied by the manufacturer (Life Technologies, Darmstadt, Germany).

### Complement hemolysis assay

A hemolysis assay was established to investigate whether Ide_*Ssuis*_ activity modulates the complement-dependent hemolysis caused by porcine Ig raised against ovine erythrocytes. For generation of sera containing these specific antibodies (αEry sera), two piglets were immunized with purified ovine erythrocytes and 10% Emulsigen (MVP Laboratories Inc., Omaha, NE). Generation of hyperimmune sera in pigs in our institute is registered under 12A226 at the Lower Saxonian State Office for Consumer Protection and Food Safety.

Sera were drawn from these piglets prior and post prime as well as post booster vaccination as indicated. Ovine erythrocytes from defibrinated blood were washed three times with 0.9% sodium chloride solution and were finally diluted to a 2% erythrocyte suspension. Hemolysis experiments were conducted with 1:20 final dilutions of porcine sera. To confirm that the hemolytic activity of the αEry sera depended on complement activity, sera were either inactivated by heat treatment (30 min 56 °C) or by addition of 10 mM EDTA (30 min 25 °C). The classical pathway was specifically inhibited by incubation of the serum with 10 mM EGTA and 15 mM MgCl_2_ (30 min 25 °C).

For functional analysis of Ide_*Ssuis*_, 1.8 μg recombinant protein of either Ide_*Ssuis*_ or a truncated derivative was incubated with 100 μL of a 1:10 dilution of porcine serum (pre immune or αEry sera) for 1.5 h on a rotator at 37 °C. Erythrocytes were mixed with treated serum (each 100 μL) and incubated for 30 min at 37 °C on a shaker. As control the erythrocytes were incubated in water (defined as complete lysis of erythrocytes) or in a 0.9% sodium chloride solution. Unlysed red blood cells were pelleted by centrifugation (1000 × *g* for 5 min). One hundred μL of the supernatant was transferred into a 96-well flat bottom microplate and the absorbance was measured at 405 nm. For the inhibition assay, 5.2 μg recombinant protein was incubated with 0.4 M iodoacetamide (or as a control PBS) for 30 min. Unbound iodoacetamide was afterwards removed using Amicon Ultra 0.5 mL centrifugal filters with a 10 kDa cut off (Merck Millipore, Schwalbach, Germany). Subsequently, the iodoacetamide-inactivated Ide_*Ssuis*_ contructs and its controls were investigated for modulation of hemolysis caused by αEry sera as described above but erythrocytes and serum were incubated for 1 h.

### Detection of IgM and IgG on the surface of ovine erythrocytes

Porcine sera drawn before and after immunization with ovine erythrocytes (see above) were inactivated by heat treatment (30 min 56 °C). A 2% erythrocyte suspension generated from EDTA-blood was incubated with inactivated pre and post immune serum. Inactivated serum was pretreated with different rIde_*Ssuis*_ constructs and as control with rMRP as described above to investigate a putative modulation of IgM and IgG binding to erythrocytes. After incubation with porcine sera, erythrocytes were centrifuged, resuspended in 5% goat serum (for the detection of IgM) or 5% rabbit serum (for the detection of IgG) and incubated on a rotator at 8 °C for 1 h. Erythrocytes were washed with PBS and incubated in a 1:250 dilution of a mouse anti-porcine IgM antibody (Serotec, Puchheim, Germany) or in a 1:10 000 dilution of a goat anti-porcine IgG antibody (Serotec) for 1 h at 8 °C. After washing of erythrocytes samples were incubated in a 1:500 dilution of a phycoerythrin-labelled goat anti-mouse IgG antibody (Bio Legend, Fell, Germany) or in a 1:1000 dilution of a Alexa fluor 488–labelled chicken anti-goat IgG antibody (Life Technologies, Darmstadt, Germany), respectively. Erythrocytes were analysed using a BD Accuri^TM^ C6 (Becton Dickinson, Heidelberg, Germany) flow cytometer. For each determination 10 000 events were acquired and analysis of erythrocytes was carried out by dot plot analysis.

### C3-deposition assay

For opsonization of *S. suis* with C3b/C3i, 150 μL serum drawn after bacterin prime-vaccination or hyperimmune serum raised against *S. suis* serotype 2 was added to 75 μL of a culture grown to an OD_600_ of 0.8. After 1 h of incubation at 37 °C under rotation, bacteria were centrifuged, washed with PBS and incubated with a polyclonal FITC-labeled rabbit anti-human C3c antibody (Dako, Eching, Germany) (1:150 diluted in PBS) for 1 h at 8 °C. For opsonization of *S. suis* 10*cps*Δ*EF* and 10*cps*Δ*EF*Δ*ideSsuis*, 75 μL serum of colostrum-deprived piglets (SCDP) with or without the addition of purified porcine IgM (0.14 mg/mL) was added to 75 μL of a culture grown to an OD_600_ of 0.8. Porcine IgM were purified as described before (8). Bacteria and serum were incubated for 30 min at 37 °C and labelled with an antibody directed against C3 as described above. Fluorescent bacteria were analysed after washing with PBS and inactivation with 0.375% formaldehyde in flow cytometry as described previously [7].

To deplete serum of complement components, serum was pretreated with zymosan as decribed [22] with the following modifications. A 225 μL aliquot of a zymosan A (Sigma, Taufkirchen, Germany) stock solution (15 mg zymosan A resuspended in 1 mL of a 14 mM sodium chloride solution) was incubated for 30 min at 100 °C. The suspension was centrifuged at 16 000 × *g* for 5 min and the pellet was resuspended in 150 μL of porcine serum, incubated for 30 min at 25 °C and centrifuged at 16 000 × *g* for 5 min. Treatment of bacteria with this supernatant was compared to treatment of bacteria with untreated serum to access the effect of complement depletion by zymosan. All three complement pathways were blocked with 10 mM EDTA (30 min 25 °C) or heat inactivation (30 min 56 °C). To inhibit only the classical complement pathway, sera were incubated with 10 mM EGTA and 15 mM MgCl_2_ for 30 min at 25 °C. The differently treated serum samples were incubated with the bacteria which were subsequently analyzed for deposition of C3 antigen as described above.

### Opsonophagocytosis assay

Opsonophagocytic killing in the presence of 20% (v/v) porcine serum was assessed essentially as described [23]. Porcine neutrophils were purified from freshly drawn blood as outlined previously [24]. To obtain a multiplicity of infection of 0.03, 1.5 × 10^5^ bacteria were added to 400 μL of a neutrophil suspension in RPMI containing 5 × 10^6^ neutrophils and 100 μL serum. The samples were incubated for 1 h at 37 °C on a rotator. Samples incubated with porcine α*S. suis* serotype 2 hyperimmune serum and serum of colostrum-deprived piglets were included as positive and negative control, respectively. The survival factor as defined by the ratio of colony forming units (CFU) at t = 60 min to the respective value at t = 0 min was determined for each strain. The ratio of the survival factors of 10Δ*ide*_*Ssuis*_ and wt was calculated to assess attenuation of the 10Δ*ide*_*Ssuis*_ mutant.

### Bactericidal assay

Survival of *S. suis* in porcine blood was determined as described in a previous study [6]. Briefly, 500 μL of heparinized blood (16 I. U. heparin/mL) were infected with 1.5 × 10^5^ CFU using stocks of frozen bacteria with 15% glycerol after thawing. The blood was incubated for 2 h at 37 °C on a rotator. Bactericidal assays were conducted with blood drawn from 5 to 7 week old piglets 6 to 14 days after prime vaccination with a *S. suis* serotype 2 bacterin. These piglets were not included in the experimental infection experiment.

### Animal experiment

German Landrace piglets (*n* = 25) free of *sly* + *mrp* + *epf* + *cps*2+ strains were infected experimentally either with strain 10 (*n* = 9) or strain 10Δ*ide*_*Ssuis*_ (*n* = 8) or 10Δ*ide*_*Ssuis*__homologue (*n* = 8). Piglets were cared for in accordance with the principles outlined in the European Convention for the Protection of Vertebrate Animals Used for Experimental and Other Scientific Purposes [25]. The animal experiment of this study was approved by the Committee on Animal Experiments of the Lower Saxonian State Office for Consumer Protection and Food Safety (permit no. 33.9-42502-04-12/0965).

All piglets were prime-vaccinated at an age of 5–6 weeks with a bacterin generated with *S. suis* strain 10 grown overnight and inactivated in 0.2% formaldehyde. Emulsigen was added as adjuvant (20% [vol/vol]). Each immunization dose contained approximately 10^9^ bacteria.

At an age of 7 to 8 weeks piglets were challenged 12 days after prime vaccination. Piglets were intranasally infected after predisposition through intranasal treatment with 1% acetic acid as described previously [16]. Criteria for morbidity were fever (≥40.2 °C) or specific clinical signs such as convulsions or severe lameness. In the case of high fever (≥40.5 °C), apathy and anorexia persisting over 36 h as well as in all cases of clinical signs of acute polyarthritis or severe meningitis animals were euthanized for reasons of animal welfare. All surviving piglets were sacrificed 15 days post infection (dpi).

After euthanasia every animal went through the same procedure of necropsy including predefined collection of samples for histological and bacteriological investigations. Fibrinous-suppurative inflammations were scored in blinded experiments as described previously [16]. To allow comparison of groups the sum of the highest scores of each animal for any of the investigated organs was divided by the number of animals (ω = Σscore_max_/n_animals_). Isolation of the challenge strains was confirmed in a PCR for detection of *epf* and *cps*2 [26] and in *ide*_*Ssuis*_-specific PCRs using oligonucleotide primers specified in Additional file 1.

### Detection of αMRP IgG as well as α*S. suis* IgM and IgG antibodies

MRP, used as antigen for the IgG ELISA, is a dominant immunogen of this *S. suis* pathotype [17,23,27]. The detection of IgG titers against MRP was performed as described [16]. For the measurement of α*S. suis* IgM or IgG antibody titers Maxisorb® plates (Nunc, Rochester, NY) were coated with 1 × 10^7^ inactivated *S. suis* wt bacteria/well. Every sample and the controls were measured in a duplicate series of four (reference serum: seven) twofold dilutions in PBST starting with a dilution of 1:50. For the detection of *S. suis* specific IgM antibodies the plates were incubated with a dilution of 1:10 000 of a POD-conjugated goat anti-porcine IgM antibody (Thermo Scientific, Schwerte, Germany, catalog number PA1-84625) for 1 h at 37 °C. Blocking, washing and development of ELISA plates as well as calculation of ELISA units was conducted as previously described [17]. Data were only considered if they met the following criteria: a deviation of duplicates of no more than 22%, a slope of the linear portion of the reference standard curve between 0.8 and 1.2, a correlation coefficient between 0.9 and 1.0, and controls within established ranges.

### Statistical analysis

Experiments were performed at least three times and if not stated otherwise one-way analysis of variance (ANOVA) using Dunnetts adjustment or Tukeys multiple comparison test was used. ELISA-values were compared using the Mann–Whitney U-Test. Statistical analysis of Kaplan-Meier diagrams was conducted with the log-rank test. Means and standard deviation of the results are shown. Probabilities lower than 0.05 were considered significant (*p* < 0.05 *, *p* < 0.01 ** and *p* < 0.001 ***).

## Results

### Ide_*Ssuis*_ cleaves the heavy chain of IgM at the N-terminus of the C3 domain

**Figure 1:**
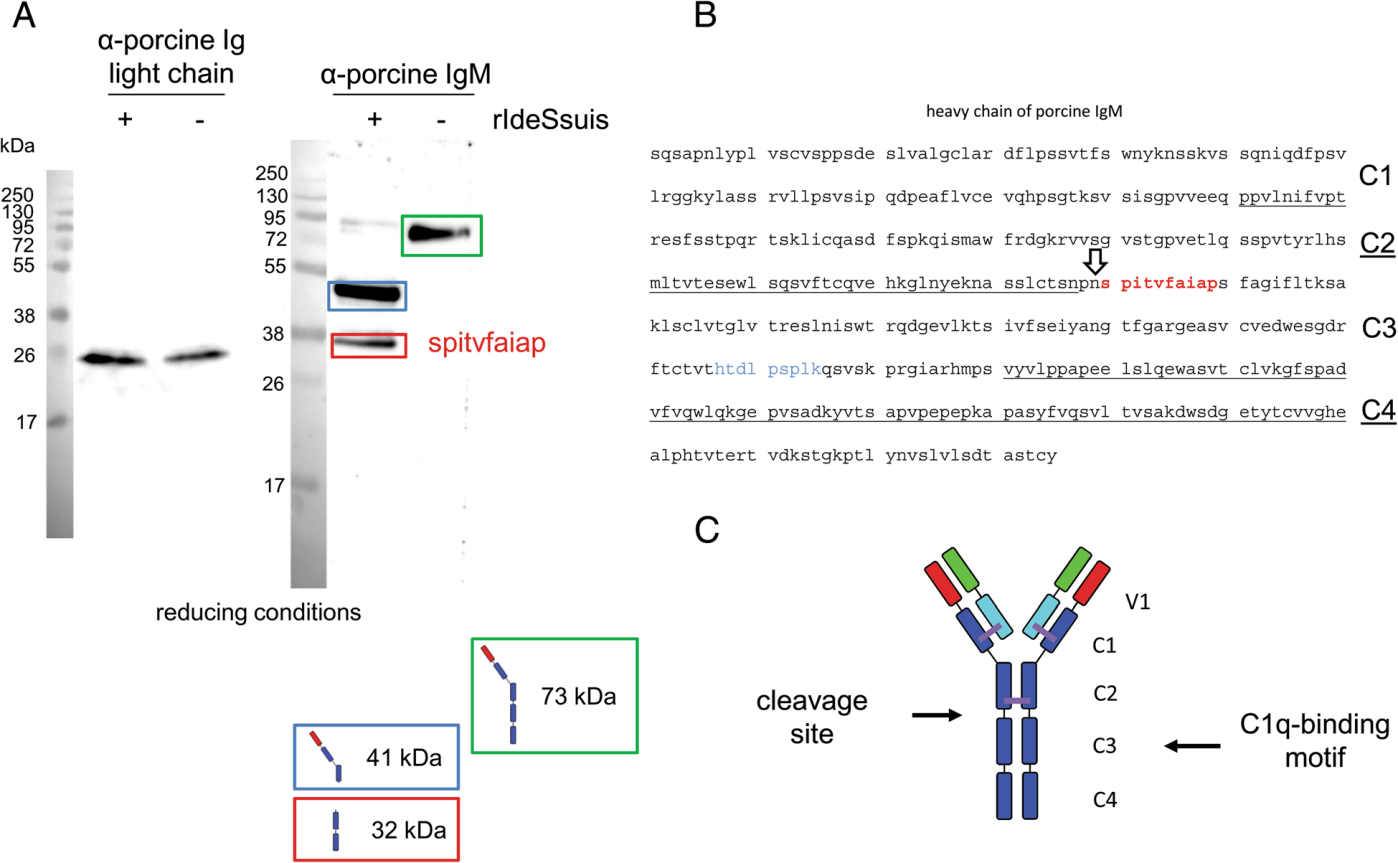
**Ide**_***Ssuis***_ **cleaves the heavy chain of porcine IgM. (A)** αIg light chain and αIgM Western blot analysis of purified porcine IgM after incubation +/− rIde_*Ssuis*_. Samples were separated in an SDS-gel under reducing conditions. The marker bands are shown on the left side (sizes in kDa). **(B)** The IgM-cleavage product highlighted in red was characterized by N-terminal Edman-sequencing. The identified sequence is shown in red and corresponds to the constant domain 3 of the IgM heavy chain. The putative C1q binding motif is marked in blue. Domains C2 and C4 are underlined. The obtained sequence was taken directly from the NCBI database. **(C)** Illustration of an IgM-monomer with the indicated cleavage site and the location of the putative C1q-binding motif.

Cleavage of IgM by Ide_*Ssuis*_ was previously identified and characterized in Western blot analysis under non-reducing conditions [6]. In this study we used a different αIgM antibody recognizing only the reduced heavy chain of IgM to detect IgM cleavage products after Ide_*Ssuis*_ incubation (Figure 1). This allowed us to successfully determine the N-terminal sequence of the 32 kDa cleavage product as SPITVFAIAP via Edman sequencing (Figure 1). Based on the N-terminal sequence, Ide_*Ssuis*_ cleaves the heavy chain of IgM at the N-terminus of the C3 domain. In accordance with this result, reducing αIgM Western blot analysis revealed two cleavage products of 41 kDa and 32 kDa, which putatively included V1-C1-C2 and C3-C4 domains of the heavy IgM chain, respectively (Noteworthy, this αIgM antibody does not recognize the light chain of IgM).

Cysteines involved in disulphide bonds are conserved between human and porcine IgM.

Assuming that constitution of disulfide bonds is also conserved between the two species, the cysteines of C1 and C2 domains of porcine IgM should form intradomain and interchain disulphide bonds but not link monomers to the multimer. The Western blot in Figure 1 and further analysis with antibodies recognizing unreduced IgM [6] and the light chain of IgM only (Figure 1A), suggested that IgM is cleaved by Ide_*Ssuis*_ only at the indicated site. IgM bound to the bacterial surface is also cleaved by Ide_*Ssuis*_ and results in release of Fcμ cleavage products [6], which most likely include the C3 and C4 domain of the heavy chain of porcine IgM. This cleavage pattern is likely to affect IgM effector functions, in particular reduced activation of the classical complement pathway, as the C3 domain includes the C1q binding motif of porcine IgM [28,29].

### Ide_*Ssuis*_ abrogates activation of the classical complement pathway

**Figure 2:**
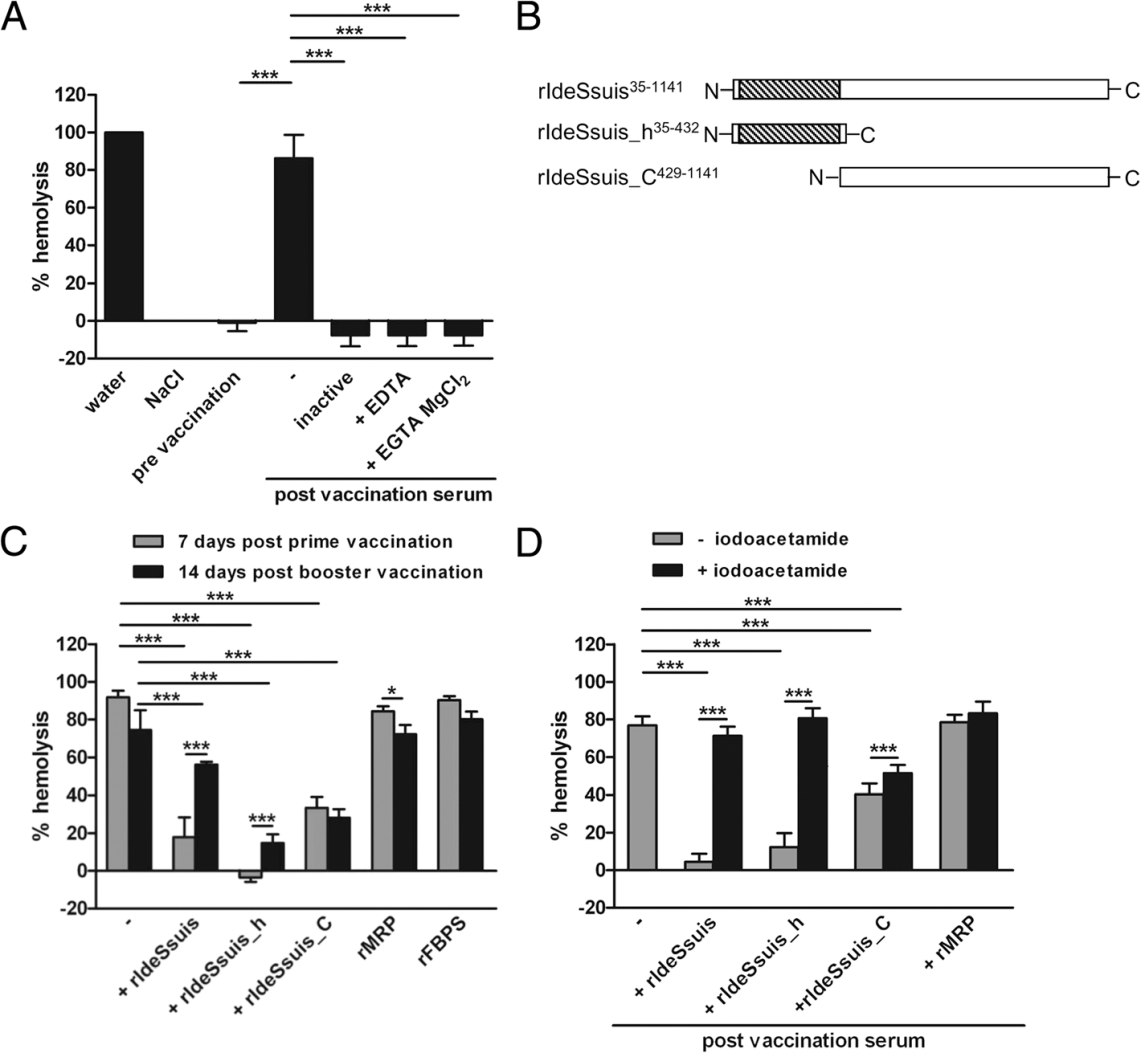
**Hemolysis caused by the classical complement activation pathway is abrogated by Ide**_***Ssuis***_ **in dependence of the protease activity. (A)** Purified sheep erythrocytes were incubated with water (defined as 100% hemolysis), physiological sodium chloride solution (NaCl), sera of a piglet drawn before (pre vaccination serum) and 7 days after prime vaccination (post vaccination αEry serum) with ovine erythrocytes. The αEry serum was heat inactivated or treated with EDTA to access the impact of complement activation. To specifically inhibit the classical complement pathway 10 mM EGTA plus 15 mM MgCl_2_ was used. **(B)** Illustration of rIde_*Ssuis*_ and its truncated derivatives. The amino acids of Ide_*Ssuis*_ included in these constructs are superscribed. The region homologous to IdeS is shaded. **(C)** Complement dependent hemolysis is reduced by pretreatment of the indicated different αEry sera with rIde_*Ssuis*_, rIde_*Ssuis*__homologue (rIdeSsuis_h) and rIde_*Ssuis*__C_terminus (rIdeSsuis_C) but not with rMRP and rFBPS. **(D)** Proteolytic activity of rIde_*Ssuis*_ and rIde_*Ssuis*__homologue is crucial for complement inhibition. The Ide_*Ssuis*_ constructs were incubated with the cysteine-protease inhibitor iodoacetamide prior to incubation with the αEry sera as indicated. The final dilutions of porcine sera in the hemolysis assay were 1:20 in all cases. Bars and error bars represent mean values and standard deviations, respectively. Significant differences are indicated (* *p* < 0.05; ** *p* < 0.01; *** *p* < 0.001).

The classical complement activation pathway can be studied in hemolysis assays using sera containing antibodies directed against erythrocytes. We investigated the impact of Ide_*Ssuis*_ on complement activation in a hemolysis assay including sera drawn from piglets vaccinated with erythrocytes (αEry sera). In accordance with complement activation, treatment of αEry sera with heat, EDTA or EGTA plus MgCl_2_ completely abolished the hemolytic activity of the αEry sera (Figure 2A). For functional analysis of Ide_*Ssuis*_, αEry sera drawn after prime and booster vaccination were treated with different rIde_*Ssuis*_ constructs (Figure 2B) prior incubation with erythrocytes. Incubation of the post-prime αEry serum with rIde_*Ssuis*_ and rIde_*Ssuis*__homologue (the domain containing the IgM protease), almost completely abolished this hemolysis (Figure 2C). Noteworthy, treatment of the post-prime αEry serum with two recombinant control proteins (MRP and FBPS), did not result in abrogation of hemolysis (Figure 2C). Interestingly, treatment of αEry sera with proteolytic inactive construct rIde_*Ssuis*__C_terminus led also to a significant reduction of hemolysis indicating a separate role of the C-terminus in complement evasion. However, significant differences between inhibition of complement activation through αEry sera drawn after prime and booster vaccination were only observed for the Ide_*Ssuis*_ constructs with IgM protease activity (Figure 2C).

The different recombinant Ide_*Ssuis*_ constructs were treated with the protease inhibitor iodoacetamide prior to incubation with αEry serum to assess the impact of proteolytic activity on complement inhibition. Preincubation of rIde_*Ssuis*_ and rIde_*Ssuis*__homologue with iodoacetamide completely abrogated the complement inhibiting activity of these proteins (Figure 2D).

**Figure 3:**
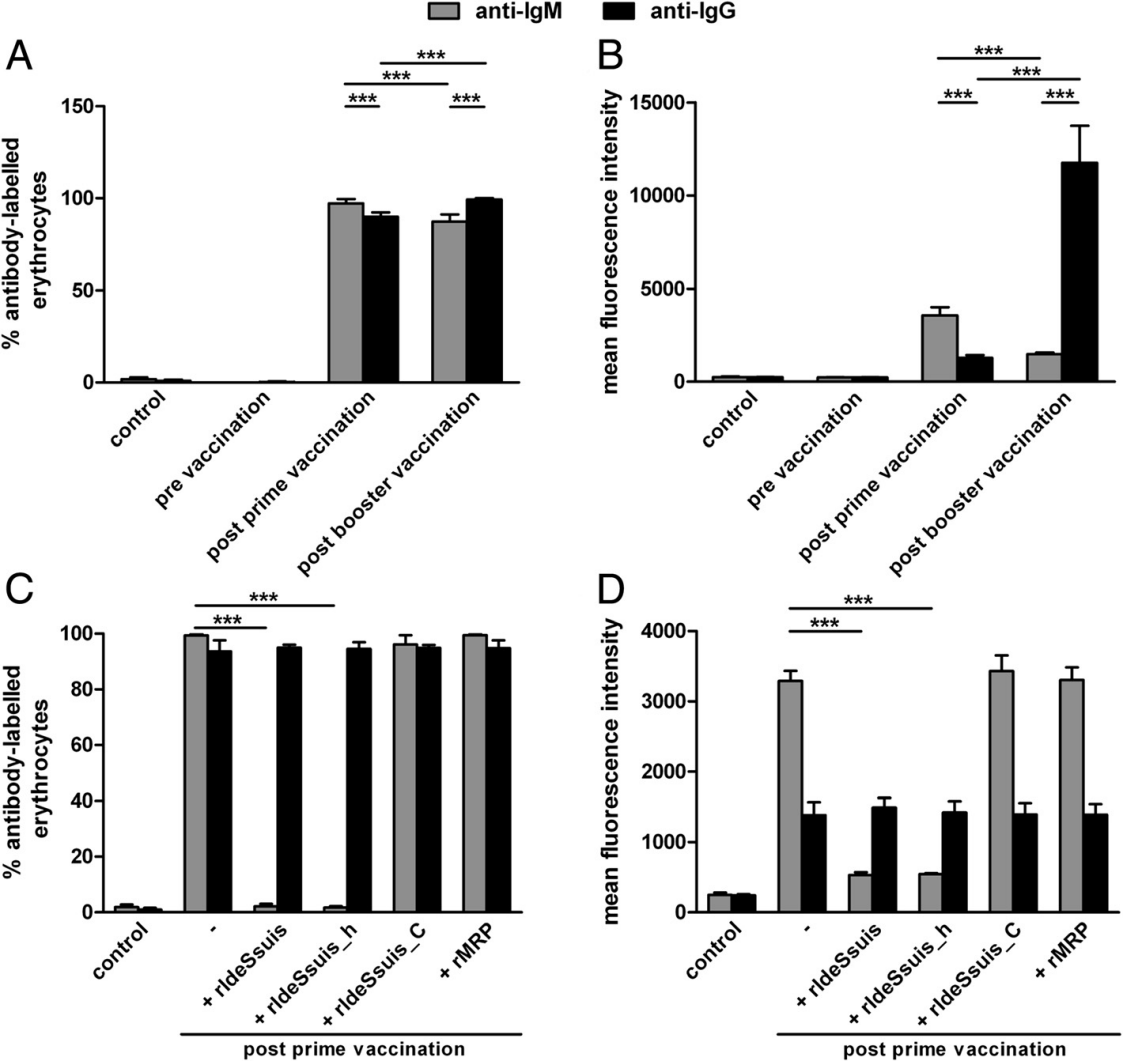
**The proteolytic rIde**_***Ssuis***_ **constructs modulate IgM binding to the surface of erythrocytes after incubation in αEry sera. (A, B)** Flow cytometry analysis of IgM and IgG binding to ovine erythrocytes after incubation with porcine sera drawn pre, post prime or post booster vaccination with ovine erythrocytes (generation of αEry sera). **(C, D)** Binding of specific IgM antigen to the surface of ovine erythrocytes is significantly reduced after pretreatment of a post prime αEry serum with rIde_*Ssuis*_ and rIde_*Ssuis*__homologue. The post prime αEry serum was preincubated with the indicated Ide_*Ssuis*_ constructs and as a control with MRP. **(A, C)** The percentage of erythrocytes labelled with IgM or IgG and **(B, D)** the mean fluorescence intensities are shown. αEry sera were inactivated in these assays to allow flow cytometric analysis of erythrocytes. Bars and error bars represent mean values and standard deviations, respectively. Significant differences are indicated (***p < 0.001) except for differences between pre and post vaccination sera.

Furthermore, flow cytometry analysis was conducted with erythrocytes after incubation with inactivated post prime and post booster αEry sera to differentiate binding of specific IgM and IgG in these sera. Both sera contained erythrocyte-specific IgG and IgM in contrast to the pre immune serum. Incubation with the post prime serum led to significantly higher percentage of IgM-labelled erythrocytes and respective mean fluorescence intensity (MFI) in comparison to the post booster serum. Vice versa, IgG staining on erythrocytes resulted in a much higher MFI after incubation in post booster serum (Figures 3A and B). We investigated modulation of IgM and IgG antigen binding to erythrocytes by treatment of the post prime αEry serum with the different recombinant Ide_*Ssuis*_ constructs. Treatment of this serum with proteolytic active rIde_*Ssuis*_ and rIde_*Ssuis*__homologue led to a significant reduction of the percentage of erythrocytes labelled with IgM and the respective MFI (Figures 3C and D) in contrast to the treatment with the non-proteolytic constructs. Binding of IgG to the erythrocytes was not modulated by incubation of the αEry serum with any of the recombinant Ide_*Ssuis*_ constructs.

In conclusion, Ide_*Ssuis*_ interferes with the classical complement activation pathway. The results of the hemolysis assay suggest that Ide_*Ssuis*_ interferes with complement activation by two mechanisms, firstly, by its IgM protease activity and, secondly, by some yet unknown function of the large non-proteolytic C-terminal domain.

### Expression of Ide_*Ssuis*_ reduces IgM-triggered complement deposition on the bacterial surface of an unencapsulated mutant

We investigated deposition of C3 on the surface of opsonized *S. suis* strains by flow cytometry to further investigate the hypothesis that Ide_*Ssuis*_ is involved in complement evasion. After opsonization of *S. suis* wt and 10Δ*ide*_*Ssuis*_ with different porcine sera with moderate to high specific IgM titers the percentage of bacteria with stained C3 antigen (most likely C3b/C3i) was slightly increased in the mutant 10Δ*ide*_*Ssuis*_ (Additional file 3). Specifically, 39.2% of wt (SD = 11.0%) and 42.8% of 10Δ*ide*_*Ssuis*_ (SD = 10.2%) bacteria were C3-labelled after opsonization in sera of bacterin-primed piglets (Additional file 3)*.* The sera of these bacterin-primed piglets had moderate to high titers of α*S. suis* IgM (34 – 103 ELISA units) and α*S. suis* IgG (69 – 161 ELISA units) but low αMRP titers (below 15 ELISA units). After addition of EGTA and MgCl_2_ to these sera only 7.1% (SD = 4.8%) and 7.7% (SD = 6.8%) of wt and 10Δ*ide*_*Ssuis*_ bacteria, respectively, were labelled with the αC3 antibody indicating that complement is mainly activated by the classical activation pathway during early adaptive immune responses (Additional file 3).

**Figure 4:**
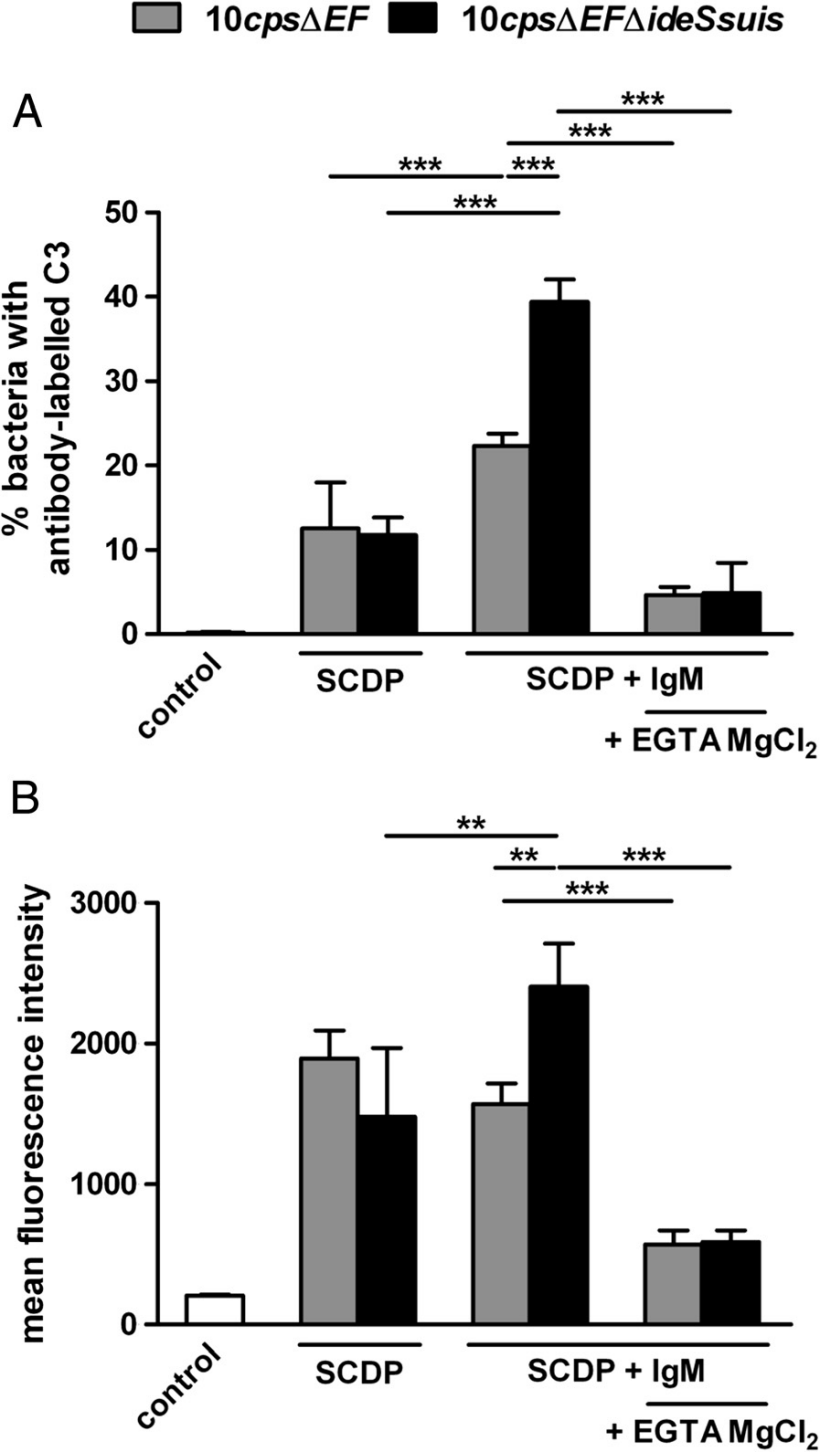
**Expression of Ide**_***Ssuis***_ **reduces C3 deposition on the surface of an unencapsulated mutant triggered by IgM-mediated activation of the classical complement pathway.** C3 antigen bound to the surface of the unencapsulated mutant 10*cps*Δ*EF* and the double mutant 10*cps*Δ*EF*Δ*ide*_*Ssuis*_ was detected through flow cytometry analysis. Bacteria were opsonized with sera from colostrum-deprived piglets (SCDP) or SCDP spiked with purified porcine IgM (SCDP plus IgM). To inactivate the classical complement activation pathway SCDP plus IgM was pretreated with EGTA MgCl_2_. As control non-opsonized bacteria (incubated in PBS) were incubated with the C3 specific antibody. **(A)** The percentage of bacteria with antibody-labelled C3 and **(B)** the mean fluorescence intensity of the samples are shown. Bars and error bars represent mean values and standard deviations, respectively. Significant differences are indicated (*, *p* < 0.05; **, *p* < 0.01; ***, *p* < 0.001).

We hypothesized that activation of the classical complement pathway in this assay was determined by specific IgM and IgG and that redundant complement evasion mechanisms in *S. suis* serotype 2 might limit detection of phenotypic differences between wt and 10Δ*ide*_*Ssuis*_. Thus, we deleted *ide*_*Ssuis*_ in an unencapsulated isogenic strain (10*cps*Δ*EF*Δ*ide*_*Ssuis*_) to avoid complement inhibition through the sialylated capsule of *S. suis* serotype 2. Furthermore, effects of specific IgG were excluded using serum from colostrum-deprived piglets (SCDP). As shown in Figure 4A approximately 10% of either 10*cps*Δ*EF* or 10*cps*Δ*EF*Δ*ide*_*Ssuis*_ bacteria were stained with C3 after opsonization with SCDP. Importantly, the percentages of C3-stained 10*cps*Δ*EF* increased to about 20% after addition of purified porcine IgM to SCDP prior to opsonization, but fourfold (to 40%) for 10*cps*Δ*EF*Δ*ide*_*Ssuis*_ bacteria lacking IgM protease activity (Figure 4). Differences in C3 deposition between 10*cps*Δ*EF* and 10*cps*Δ*EF*Δ*ide*_*Ssuis*_ were highly significant for the percentage of labelled bacteria and the MFI (Figure 4). Addition of the classical complement pathway inhibitor (EGTA plus MgCl_2_) reduced the percentage of C3 labelled bacteria to 4.7% (10*cps*Δ*EF*) and 4.9% (10*cps*Δ*EF*Δ*ide*_*Ssuis*_) and diminished the phenotype of the double mutant. These results confirm that C3 deposition on the surface of *S. suis* might be determined by IgM-mediated activation of the classical complement pathway and show that *S. suis* reduces this IgM-mediated C3 deposition by expression of Ide_*Ssuis*_.

### The mutant 10Δ*ide*_*Ssuis*_ is attenuated in survival in opsonophagocytosis assays in the presence of specific IgM

**Figure 5:**
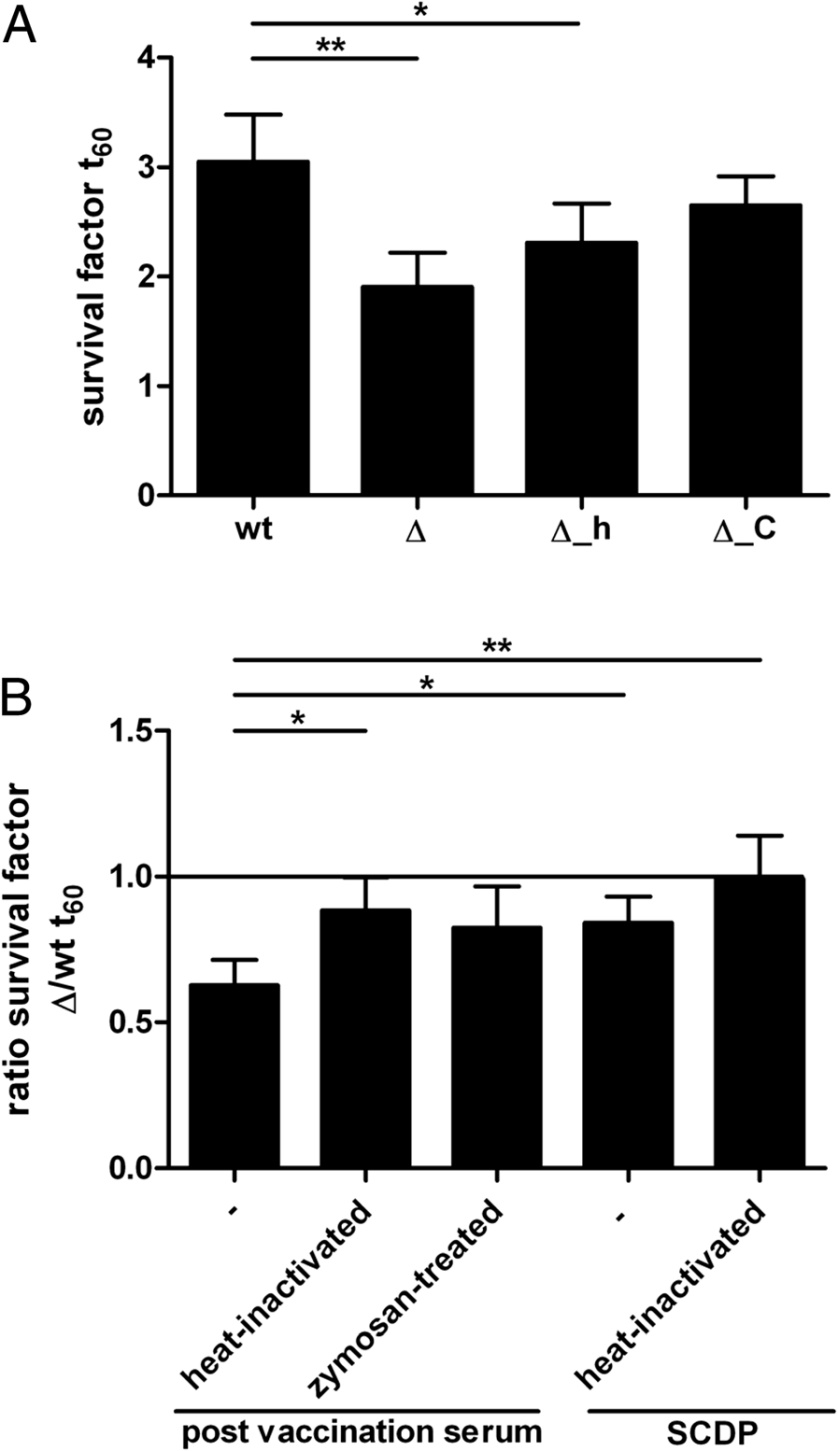
**Ide**_***Ssuis***_ **promotes survival in opsonophagocytosis assays including purified porcine neutrophilic granulocytes and serum with specific IgM titers. (A)** Survival of strain 10 (wt), 10Δ*ide*_*Ssuis*_ (Δ), 10Δ*ide*_*Ssuis*__homologue (Δ_h) and 10Δ*ide*_*Ssuis*__C-terminus (Δ_C) in opsonophagocytosis assays with serum from a piglet with specific IgM. **(B)** Attenuation of 10Δ*ide*_*Ssuis*_ in opsonophagocytosis assays depends on active complement and adaptive immunity. The ratios of the survival factors of 10Δ*ide*_*Ssuis*_ to the respective survival factors of wt are shown. A ratio of 1 is depicted by the horizontal line and refers to a lack of attenuation. To access the impact of complement, the post immune serum with specific IgM used for experiments shown in **(A)** was heat-inactivated or treated with zymosan. Samples with active and heat-inactivated serum from a colostrum-deprived pig were included to investigate whether this Ide_*Ssuis*_-mediated phenotype depends on adaptive immunity. Bars and error bars represent mean values and standard deviations, respectively. Significant differences between strains and ratios of survival factors in **(A)** and **(B)**, respectively, are indicated (* *p* < 0.05, ** *p* < 0.01).

As rIde_*Ssuis*_ interfered with complement activation using sera with specific antibodies, we hypothesized that expression of the IgM protease Ide_*Ssuis*_ contributes to survival in opsonophagocytosis assays including a porcine serum with specific IgM and comparatively low specific IgG titers (α*S. suis* IgM: 29.2 ELISA-units and for comparison: αMRP IgG: 12.8 ELISA-units). Phenotypic analysis of *S. suis* was conducted in this study using the mutant 10Δ*ide*_*Ssuis*_ [6] and two new in frame deletion mutants expressing truncated Ide_*Ssuis*_ constructs. These new mutants, designated 10Δ*ide*_*Ssuis*__C-terminus and 10Δ*ide*_*Ssuis*__homologue, expressed the N-terminal part homologous to IdeS and the large C-terminal part lacking homologies, respectively (Additional file 4). Noteworthy, 10Δ*ide*_*Ssuis*_ _C-terminus released IgM protease activity in the supernatant in contrast to 10Δ*ide*_*Ssuis*_ and 10Δ*ide*_*Ssuis*__homologue (Additional file 4). As shown in Figure 5A 10Δ*ide*_*Ssuis*_ and 10Δ*ide*_*Ssuis*__homologue had a significant lower survival factor compared to the wt strain and the survival factor for 10Δ*ide*_*Ssuis*__C-terminus was also found to be lower compared to the wt strain. The extent of attenuation of 10Δ*ide*_*Ssuis*_ was significantly lower in opsonophagocytosis assays including serum from a colostrum-deprived piglet in comparison to assays including specific IgM. Inhibition of complement reduced the attenuation of the mutant 10Δ*ide*_*Ssuis*_ significantly (Figure 5B).

### Experimental infection of prime-vaccinated growing piglets suggests attenuation of the mutant 10Δ*ide*_*Ssuis*_

**Figure 6:**
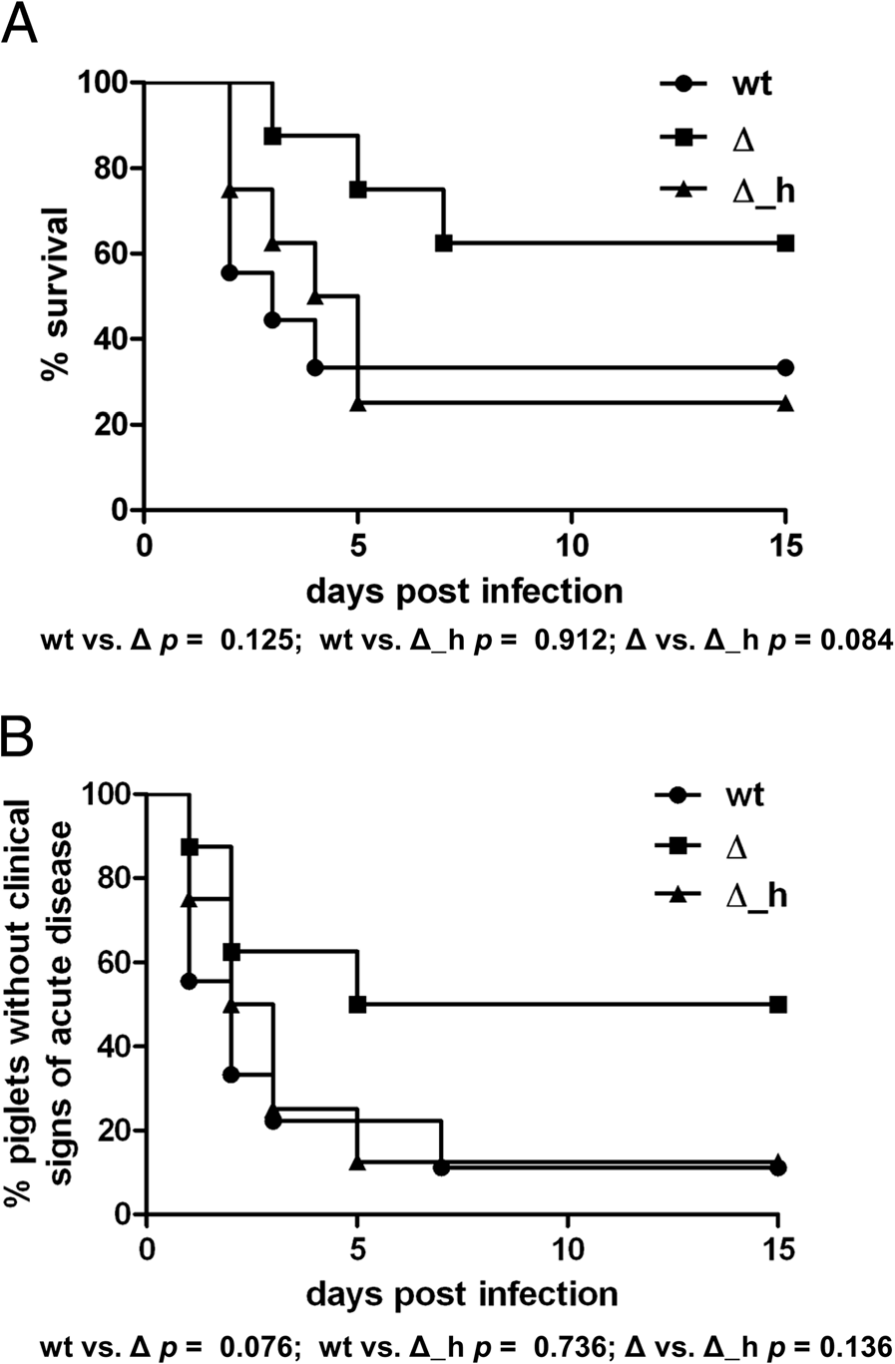
**Mortality (A) and morbidity (B) of prime-vaccinated growing piglets intranasally challenged with the indicated** ***S. suis*** **strains.** Growing piglets were infected with wild type strain 10 (wt), 10Δ*ide*_*Ssuis*_ (Δ) and 10Δ*ide*_*Ssuis*_ _homologue (Δ_h) 12 days after prime vaccination with a homologous *S. suis* serotype 2 bacterin. A piglet was determined as morbid in the case of elevated body temperature (≥40.2 °C) or specific clinical signs (signs of central nervous dysfunction or severe lameness). Statistical analysis of the Kaplan-Meier diagrams was conducted with the log-rank test (all *p*-values are shown below the diagrams).

Based on the in vitro results we considered Ide_*Ssuis*_ to be a putative virulence factor of *S. suis* in piglets with high titers of specific IgM. Thus, we conducted experimental infection of piglets prime vaccinated with a bacterin. Immunological screening of these piglets confirmed that these piglets had high specific IgM titers and low IgG titers against MRP, a main immunogen of this invasive *S. suis* pathotype (Additional file 5). We infected piglets with the wt and the 10Δ*ide*_*Ssuis*_ mutant as well as with the partial mutant 10Δ*ide*_*Ssuis*__homologue, which expressed only the C-terminus and showed no IgM proteolysis (Additional file 4). The complemented mutant was not included in the experimental infection because it showed attenuation in growth in medium (unpublished results). Sixty three percent of the piglets infected with the mutant 10Δ*ide*_*Ssuis*_ survived this experiment, whereas only 33% did so in the wt infected group (*p* = 0.125) (Figure 6A). Furthermore, 50% and 11% of 10Δ*ide*_*Ssuis*_ and wt infected piglets*,* respectively, were free of clinical signs throughout the observation period (*p* = 0.076; Figure 6B). Furthermore, 10Δ*ide*_*Ssuis*_ infected piglets had a lower pathohistological score (ω = 2.2) in comparison to wt infected animals (ω = 3.7; Table 1). In general, detection of fibrinosuppurative lesions was associated with detection of the infection strain. The mutant 10Δ*ide*_*Ssuis*_ was not detectable in any inner organ in 5 of 8 infected piglets (Table 2). However, the group infected with the deletion mutant 10Δ*ide*_*Ssuis*__homologue showed mortality and morbidity as well as a high rate of infection of inner organs very similar to the wt infected group (Figure 6; Table 2).

**Table 1:** **Scoring of fibrinosuppurative lesions of growing piglets intranasally infected with the indicated** ***S. suis*** **strains after prime-vaccination with a** ***S. suis*** **serotype 2 bacterin**

**Infection strain** ^**a**^	**Piglets without lesions**	**Piglets with lesions in three or more locations**	**Brain**			**Serosae**			**Joint**			**Spleen and liver**			**Lung**			**Heart**			
			**Meningitis, chorioiditis**			**Pleuritis or peritonitis**			**Synovialitis**			**Splenitis** ^**b**^ **or hepatitis**			**Pneumonia**			**Endocarditis**			
			**5** ^**c**^	**3** ^**d**^	**1** ^**e**^	**4** ^**c**^	**2** ^**d**^	**1** ^**e**^	**4** ^**c**^	**2** ^**d**^	**1** ^**e**^	**4** ^**c**^	**2** ^**d**^	**1** ^**e**^	**4** ^**c**^	**2** ^**d**^	**1** ^**e**^	**4** ^**c**^	**2** ^**d**^	**1** ^**e**^	***ω*** ^***f***^
wt	1/9	5/9	1/9	0/9	0/9	4/9	0/9	0/9	2/9	0/9	0/9	3/9	3/9	0/9	6/9	0/9	0/9	0/9	0/9	0/9	3.7
Δ	3/8	3/8	3/8	0/8	0/8	0/8	1/8	0/8	1/8	2/8	0/8	1/8	1/8	3/8	1/8	2/8	0/8	0/8	0/8	0/8	2.2
Δ_h	2/8	4/8	2/8	0/8	0/8	3/8	0/8	0/8	0/8	0/8	0/8	0/8	4/8	2/8	3/8	1/8	0/8	2/8	0/8	0/8	2.9

^a^Infection strains were strain 10 (wt), 10Δ*ide*_*Ssuis*_ (Δ) and 10Δ*ide*_*Ssuis*_ _homologue (Δ_h).

^b^Neutrophilic accumulation of the splenic red pulp.

^c^Scoring of 4 and 5 indicates moderate to severe diffuse or multifocal fibrinosuppurative inflammations.

^d^Scoring of 2 and 3 indicates mild focal fibrinosuppurative inflammation.

^e^Individual single perivascular neutrophils received a score of 1.

^f^ω = Σscore_max_/n_animals_ [15].

**Table 2:** **Reisolation of the infection strain from pigs primed with a bacterin and then infected with the indicated strains**

**Infection strain** ^**a**^	**Number of pigs with an isolate of the infection strain in at least one inner organ** ^**b**^	**Number of pigs with indicated site of infection strain** ^**a**^ **isolation/total number of pigs**							
		**Tonsils**	**Lung** ^**c**^	**Serosa** ^**d**^	**Spleen**	**Liver**	**Brain, CSF** ^**e**^	**Joint fluid** ^**f**^	**Endocard**
wt	6/9	5/9	5/9	5/9	5/9	6/9	1/9	3/9	3/9
Δ	3/8	2/8	1/8	0/8	3/8	2/8	3/8	1/8	1/8
Δ_h	7/8	3/8	6/8	5/8	6/8	5/8	3/8	5/8	6/8

^a^Infection strains were strain 10 (wt), 10Δ*ide*_*Ssuis*_ (Δ) and 10Δ*ide*_*Ssuis*_ _homologue (Δ_h). Identification was conducted through PCR as described in Materials and methods.

^b^Piglets with isolates of the challenge strain exclusively from the tonsil were not considered.

^c^One cranial lobe was investigated.

^d^Pleural, peritoneal or pericardial cavity.

^e^Cerebrospinal fluid.

^f^Punctures of both tarsal and carpal joints were investigated in each animal. In cases of lameness additional joint punctures of the respective limb were screened.

In summary, the results of the experimental infection suggested an attenuation of the mutant 10Δ*ide*_*Ssuis*_ in prime-vaccinated growing piglets with high titers of specific IgM.

### Ide_*Ssuis*_ positively affects survival of *S. suis* in blood of piglets with high specific IgM titers ex vivo

**Figure 7:**
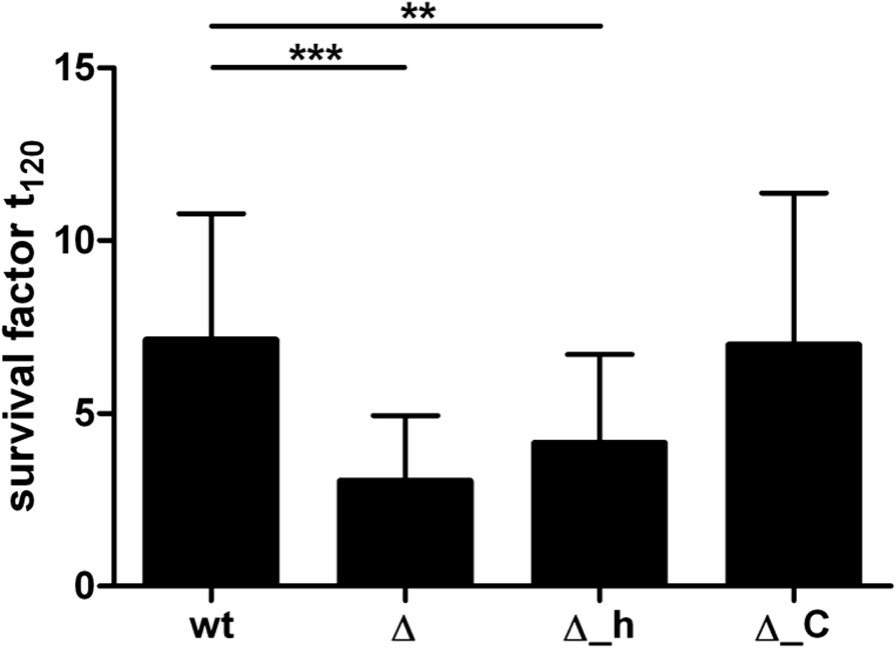
**The mutants 10Δ** ***ide***_***Ssuis***_ **(Δ) and 10Δ** ***ide***_***Ssuis***_ **_homologue (Δ_h) are attenuated in survival in porcine blood ex vivo.** The blood was drawn from 7 different growing piglets with an age of 5–7 weeks 6–14 days after prime vaccination with a *S. suis* bacterin. The mutant 10Δ*ide*_*Ssuis*_ _C-terminus (Δ_C) not deficient in IgM proteolysis was also included. The bars and error bars represent mean values and standard deviations, respectively. Significant differences are indicated (** *p* < 0.01; *** *p* < 0.001).

As bacteremia is considered to be a critical step in the pathogenesis of invasive *S. suis* diseases, we further investigated survival of the different *ide*_*Ssuis*_ mutants and the wt in porcine blood with high IgM titers ex vivo. Thus, we evaluated Ide_*Ssuis*_-dependent survival in blood from bacterin prime-vaccinated piglets. These piglets had significantly higher IgM titers against *S. suis* than unvaccinated weaning piglets investigated for comparison (*p* < 0.01; Additional file 6). As shown in Figure 7, the two mutants deficient in IgM proteolysis (10Δ*ide*_*Ssuis*_ and 10Δ*ide*_*Ssuis*__homologue) are significantly attenuated in survival in blood drawn from piglets prime-vaccinated with a bacterin. In contrast, the mutant expressing the truncated N-terminal domain with IgM protease activity (10Δ*ide*_*Ssuis*__C-terminus) was not attenuated in growth in porcine blood ex vivo.

In conclusion, expression of the IgM protease Ide_*Ssuis*_ promotes increased survival of *S. suis* in porcine blood ex vivo, at least in the presence of specific IgM.

## Discussion

The IgM pentamer is a very important activator of the classical complement pathway. It has been estimated that the efficiency of one pentameric IgM molecule to activate complement is equivalent to the respective efficiency of 1000 IgG molecules [29]. As cleavage of IgM by Ide_*Ssuis*_ occurs at a site located between the antigen-recognizing part and the Fc-part containing the putative C1q binding motif, it was reasonable to hypothesize that IgM cleavage by Ide_*Ssuis*_ is an important complement evasion mechanism of *S. suis*. In this study we obtained in vitro data supporting the hypothesis that Ide_*Ssuis*_ is involved in complement evasion: (i) different recombinant Ide_*Ssuis*_ constructs abolished the hemolysis induced by activation of the classical complement pathway in serum with specific IgM directed against erythrocytes; (ii) attenuation of the mutant 10Δ*ide*_*Ssuis*_ in opsonophagocytosis assays was complement-dependent; (iii) IgM-triggered deposition of C3 on the bacterial surface is reduced by Ide_*Ssuis*_ expression and (iv) attenuation of 10Δ*ide*_*Ssuis*_ in survival in porcine blood ex vivo was observed in blood from piglets with high specific IgM titers.

The hemolysis assays of this study showed that Ide_*Ssuis*_ interferes substantially with activation of the classical complement pathway. Importantly, Ide_*Ssuis*_ mediated inhibition of complement activation by erythrocyte specific IgM was abrogated by pretreatment of Ide_*Ssuis*_ and Ide_*Ssuis*__homologue with the protease inhibitor iodoacetamide. As IgM is the only known substrate of this protease [6] and inhibition of IgM proteolysis by iodoacetamide was confirmed in this assay, we conclude that IgM proteolysis is involved in interference of Ide_*Ssuis*_ with complement activation. Accordingly, C3 deposition on 10*cps*Δ*EF*Δ*ide*_*Ssuis*_ bacteria in serum from colostrum-deprived piglets spiked with specific IgM is significantly higher compared to C3 deposition on the Ide_*Ssuis*_ expressing strain 10*cps*Δ*EF*.

Though IgM is much more powerful in activation of the classical complement pathway than IgG, immune evasion mechanism of bacteria counteracting the classical complement pathway have so far only been described for factors interacting with IgG. Specifically, protein H expressed by group A streptococci (GAS) reduces C3 deposition on IgG-coated beads and inhibits immune hemolysis of IgG-sensitized erythrocytes [30]. Furthermore, protection against opsonophagocytic killing of GAS in the presence of specific IgG is mediated by M-proteins and M-like proteins acting as Fc-receptors [31] and by the IgG protease IdeS [32]. In light of the different virulence factors counteracting IgG-mediated activation of the classical complement pathway, it is very much surprising that a bacterial evasion mechanism counteracting IgM-mediated complement activation has to the best of our knowledge not been described. The interference of Ide_*Ssuis*_ with IgM-mediated complement activation is important for pathogenesis, since survival of *S. suis* in porcine blood of prime-vaccinated piglets is significantly increased by Ide_*Ssuis*_ expression and clinical as well as pathological findings after experimental infection of respective piglets suggested attenuation of the isogenic mutant 10Δ*ide*_*Ssuis*_.

Deposition of C3b on the surface of the encapsulated *S. suis* serotype 2 strains was only slightly determined by Ide_*Ssuis*_ expression under the chosen experimental conditions. The percentage of bacteria with detectable C3b deposition was below 16% after incubation of *S. suis* strains 10 and 10Δ*ide*_*Ssuis*_ in serum of colostrum-deprived piglets supplemented with purified porcine IgM (results not shown) in contrast to the results shown for the unencapsulated mutants (Figure 4). This indicates in accordance with published results [7] that the capsule of serotype 2 is a main inhibitor of C3b deposition. Thus, we speculate that Ide_*Ssuis*_ expression might be crucial for bacterial survival (i) during reduced capsule expression, (ii) in the presence of very high IgM titers against the polysaccharide capsule and (iii) in strains of serotypes that lack capsular sialic acid [8].

Two factor H binding proteins, have recently been identified in *S. suis* [12,13]. Factor H bound to the surface of *S. suis* serves as a cofactor for the factor-I mediated cleavage of C3b [12]. Deletion of the gene *fhb* encoding one of the factor H binding proteins led to a significant increase in C3b/iC3b deposition after opsonization with human serum. Activation of human complement was elicited by *S. suis* mainly via the alternative pathway under the chosen experimental conditions. However, in this work we demonstrate that in growing piglets with high titers of specific IgM the percentage of bacteria with antibody-labelled C3 is mainly determined by the classical pathway. This is important, because piglets with early adaptive immune responses are often affected by *S. suis* diseases and development of vaccines eliciting protection during this early immune response stage would substantially improve animal health.

Cleavage of IgM might have important biological consequences in addition to prevention of C3b deposition on the bacterial surface. Based on the identified cleavage site in the IgM heavy chain, Ide_*Ssuis*_ activity should lead to release of a pentameric Fc-molecule including only C3 and C4 domains of the heavy IgM chains. We speculate that the cleavage product detected above the 250 kDa marker lane in Western blot analysis under non-reducing conditions [6] constitutes this pentameric molecule. Future studies should consider whether this putative C3-C4-pentamer modulates functions of the immune system. It is known that ½Fc IgG fragments released upon cleavage of IgG by IdeS prime neutrophils to respond to a second stimulus with an enhanced rate of reactive oxygen species production [33]. This might lead to activation of immune cells at sites remote from the pathogen. Similarly, the putative C3-C4 pentamer might also activate immune cells, e.g. by binding to the Fcμ receptor [34].

The results of the experimental infection suggested attenuation of the mutant 10Δide_*Ssuis*_. However, the mutant expressing only the C-terminus of Ide_*Ssuis*_ (10Δ*ide*_*Ssuis*__homologue) caused morbidity in prime-vaccinated piglets comparable to the wt and unlike the mutant 10Δ*ide*_*Ssuis*_, which suggests that IgM proteolysis as such was not crucial for the outcome of the animal experiment and that the C-terminus of Ide_*Ssuis*_ carries out important, yet unknown functions. Accordingly, the recombinant truncated protein consisting only of the C-terminus of Ide_*Ssuis*_ showed also a significant interference with complement activation in the hemolysis assay suggesting an additional function of Ide_*Ssuis*_ in complement inhibition but IgM proteolysis. Interestingly, the interference of this non-proteolytic construct did not seem to depend on the ratio of erythrocyte-specific IgM and IgG titers, in contrast to the interference by the IgM protease domain (as estimated by comparative analysis of post prime and post booster αEry sera). Further studies are certainly needed to decipher further functions of Ide_*Ssuis*_ in complement evasion and their role in host-pathogen interaction.

Host-pathogen interaction of *S. suis* was investigated during early adaptive immune responses in this study. Survival in porcine blood with high specific IgM titers is significantly determined by expression of the IgM protease Ide_*Ssuis*_. Accordingly, the in vitro results of this study demonstrate that Ide_*Ssuis*_ abrogates activation of the classical complement system. As Ide_*Ssuis*_ is expressed by all investigated *S. suis* strains [6], this unique virulence mechanism appears to be crucial for the evolutionary success of this pathogen.

## Additional files

Additional file 1:
**Sequences of oligonucleotide primers.** Name, sequence and position of target sequence of primers used in this study. Additional file 2:
**Antibodies used in Western blot analysis.** Specificity, source, conjugation and used dilution of antibodies. Additional file 3:
**Flow cytometry analysis of C3 antigen (C3b/C3i) bound to opsonized** ***S. suis*** **strain 10 (wt) and 10**Δ***ide***_***Ssuis***_ **(**Δ**).** Mean values and standard deviations (S. D.) for the percentage of C3-labelled bacteria and respective mean fluorescence intensity after opsonization of bacteria with sera including specific immunoglobulins as indicated. Additional file 4:
**10**Δ***ide***_***Ssuis***_ ***_*** **homologue (**Δ**_h) and 10**Δ***ide***_***Ssuis***_ ***_*** **C-terminus (**Δ**_C) release stabile fragments of Ide**_***Ssuis***_ **into the supernatant in accordance with IgM cleavage activity in the case of** Δ**_C.** (A) αIde_Ssuis__C-terminus and αIde_Ssuis__homologue Western blot analysis of culture supernatants of 10Δ*ide*_*Ssuis*_ _homologue (Δ_h), 10Δ*ide*_*Ssuis*__C-terminus (Δ_C), 10Δ*ide*_*Ssuis*_ (Δ) and wild type strain 10 (wt). (B) 10Δ*ide*_*Ssuis*__C-terminus but not 10Δ*ide*_*Ssuis*_ *_*homologue exhibits IgM-cleaving activity. αIgM Western blot analysis of diluted porcine serum incubated with concentrated culture supernatants of 10Δ*ide*_*Ssuis*__homologue (Δ _h), 10Δ*ide*_*Ssuis*_ _C-terminus (Δ_C), 10Δ*ide*_*Ssuis*_ (Δ) and wild type strain 10 (wt) or with PBS. Additional file 5:
**Immunological analysis of piglets used in the experimental infection (see Figure** 6 **).** (A) α-*S. suis* serotype 2 (ST2) IgM, (B) α-ST2 IgG titers and (C) α-MRP IgG titers were determined in serum samples of piglets after prime vaccination with a *S. suis* ST2 bacterin (before experimental infection) and as a control in unvaccinated piglets. Significant differences are indicated. Mean values are shown by horizontal lines. Additional file 6:
**Immunological analysis of piglets used for the bactericidal assay (see Figure** 7 **).** (A) α-*S. suis* serotype 2 (ST2) IgM and (B) α-MRP IgG titers were determined in serum samples of these 7 growing piglets (prime-vaccinated) and for comparison in unvaccinated piglets. Prime vaccination was conducted with a *S. suis* serotype 2 bacterin. Significant differences are indicated (** *p* < 0.01). Horizontal lines represent mean values.

## Abbreviations

*S.*: *Streptococcus*
Ide_*Ssuis*_: Immunoglobulin M-degrading enzyme of *S. suis*
Ig: Immunglobulin
*E.*: *Escherichia*
MRP: Muramidase-released protein
FBPS: Fibronectin-and fibrinogen-binding protein of *S. suis*
TBST: Tris-buffered saline plus 0.1% Tween 20
PBST: PBS plus 0.1% Tween 20
SCDP: Serum of colostrum-deprived piglets
MFI: Mean fluorescence intensity
CFU: Colony forming units
Dpi: Days post infection
GAS: Group A streptococci
Fhb: Factor H binding protein
ST: Serotype
CSF: Cerebrospinal fluid
