# Supplementary material for: The immunoglobulin M-degrading enzyme of Streptococcus suis, IdeSsuis, is involved in complement evasion
Source: Vet Res. 2015 Apr 19;46(1):45. doi: 10.1186/s13567-015-0171-6 (PMC4404118; doi:10.1186/s13567-015-0171-6)
Supplement: Additional file 1: — Sequences of oligonucleotide primers. Name, sequence and position of target sequence of primers used in this study. [file 13567_2015_171_MOESM1_ESM.pdf]

primer	sequence	position[a]
preProIde$_{Ssuis}$PstI	CTTCTGCAGTAAAAGACGCATG	-504 to - 483
postSSide$_{Ssuis}$BamHI	ATTGGATCCTGTAACAACTGTATCGGC	94 to 114
preEndide$_{Ssuis}$BamHI	TAAGGGATCCTCAAGACAGATTATGGGAGT	3346 to 3365
postEndide$_{Ssuis}$EcoRI	AACGGAATTCTATACTTCAACTTATTCAC	3926 to 3945
Ide$_{Ssuis}$delh_for_BamHI	TTTCGGATCCCTTGCAAAAGCCAAAGAAAC	1291 to 1310
Ide$_{Ssuis}$delh_rev_SacI	AAGGAGCTCTTCAACTTGGGCTACC	1878 to 1898
ide$_{Ssuis}$delCforPstI	CTACTGCAGAACGGACTTACGGCGCTAT	702 to 720
ide$_{Ssuis}$delCrevBamHI	TGTGGATCCGGCTTTTGCAAGCGGATTG	1284 to1302
ide$_{Ssuis}$delCforBamHI	TTAGGATCCTTAAAGAAAAGACGTAAGTA	3406 to 3425
ide$_{Ssuis}$delCrevEcoRI	GCTGAATTCATGCCTGATTT	3989 to 4008

[a]. Numbers indicate the location of the oligonucleotide primer with regard to the initiation ATG codon. The gene *ide*$_{Ssuis}$ ends at position 3426.
