# Supplementary material for: The immunoglobulin M-degrading enzyme of Streptococcus suis, IdeSsuis, is involved in complement evasion
Source: Vet Res. 2015 Apr 19;46(1):45. doi: 10.1186/s13567-015-0171-6 (PMC4404118; doi:10.1186/s13567-015-0171-6)
Supplement: Additional file 2: — Antibodies used in Western blot analysis. Specificity, source, conjugation and used dilution of antibodies. [file 13567_2015_171_MOESM2_ESM.pdf]

detection of	first antibody				second antibody			
	specificity	source	conjugation	dilution	specificity	source	conjugation	dilution
porcine IgM	anti-porcine IgM µ chain (Lifespan Biosciences, Seattle, USA, LS-C59959)	rabbit PAB[a]	POD[3]	1:2000				
porcine IgM	anti-porcine IgM (Bethyl Laboratories, Montgomery, USA A100-100P)	goat PAB[a]	-	1:8000	anti-goat IgG (Jackson Laboratories, Bar Harbor, USA)	rabbit	POD[c]	1:10000
porcine Ig light chain	anti-porcine Ig lambda light chain Serotec, Duesseldorf, Germany, MCA633)	mouse MAB[b]	-	1:2000	anti-mouse IgG (GE Healthcare Freiburg, Germany)	sheep	POD[c]	1:10000
Ide_{Ssuis}	$rIde_{Ssuis}$	rabbit PAB[a]	-	1:1000	anti-rabbit IgG (GE Healthcare, NA-934)	donkey	POD[c]	1:10000
	$rIde_{Ssuis}$_homologue	rabbit PAB[a]	-	1:1000	anti-rabbit IgG (GE Healthcare, NA-934)	donkey	POD[c]	1:10000
	$rIde_{Ssuis}$_C_domain	rabbit PAB[a]	-	1:1000	anti-rabbit IgG (GE Healthcare, NA-934)	donkey	POD[c]	1:10000

[a] polyclonal antibody

[b] monoclonal antibody

[c] peroxidase
