# Supplementary material for: The immunoglobulin M-degrading enzyme of Streptococcus suis, IdeSsuis, is involved in complement evasion
Source: Vet Res. 2015 Apr 19;46(1):45. doi: 10.1186/s13567-015-0171-6 (PMC4404118; doi:10.1186/s13567-015-0171-6)
Supplement: Additional file 3: — Flow cytometry analysis of C3 antigen (C3b/C3i) bound to opsonized S. suis strain 10 (wt) and 10Δide Ssuis (Δ). Mean values and standard deviations (S. D.) for the percentage of C3-labelled bacteria and respective mean fluorescence intensity after opsonization of bacteria with sera including specific immunoglobulins as indicated. [file 13567_2015_171_MOESM3_ESM.docx]

|  |  | % C3-labelled bacteria (S.D.) | | | | |  | mean fluorescence intensity (S.D.) | | | | |
| --- | --- | --- | --- | --- | --- | --- | --- | --- | --- | --- | --- | --- |
|  |  | moderate IgM serum^a^ | |  | high IgM sera^b^ | |  | moderate IgM serum^a^ | |  | high IgM sera^b^ | |
|  |  | wt | Δ |  | wt | Δ |  | wt | Δ |  | wt | Δ |
| -^c^ |  | 0.1  (±0.1) | - |  | 0.3  (±0.1) | - |  | 168  (±6) | - |  | 191  (±8) | - |
| active |  | 64.4  (±8.2) | 69.2  (±7.6) |  | 39.2  (±11.0) | 42.8  (±10.2) |  | 2046  (±111) | 2168  (±222) |  | 1702  (±277) | 1820  (±250) |
| inactive^d^ |  | 1^#^  (±0.6) | 0.3^§^  (±0.1) |  | - | - |  | 208^#^  (±16) | 184^§^  (±7) |  | - | - |
| + EDTA |  | 1.2^#^  (±0.9) | 0.1^§^  (±0.1) |  | - | - |  | 214^#^  (±34) | 173^§^  (±3) |  | - | - |
| + zymosan |  | 3.7^#^  (±1.2) | 5.6^§^  (±1.9) |  | - | - |  | 234^#^  (±15) | 256^§^  (±20) |  | - | - |
| + EGTA MgCl_2_ |  | 48.7^#^  (±7.0) | 50.1^§^  (±4.9) |  | 7.1^#^  (±4.8) | 7.7^§^  (±6.8) |  | 2198  (±297) | 2096  (±549) |  | 1224  (±651) | 1322  (±857) |

^a^ serum with moderate specific IgM titers and low specific IgG titers (α*S. suis* IgM: 29.2 ELISA units, αMRP IgG: 12.8 ELISA units; α*S. suis* IgG: 46.1 ELISA units)

^b^ sera of 5 different prime-vaccinated growing piglets with high IgM titers against *S. suis* and low or no detectable specific IgG titers (α*S. suis* IgM: 34-103 ELISA units, αMRP IgG: not detectable - 13 ELISA units, α*S. suis* IgG: 69 – 161 ELISA units)

^c^ control, wt was incubated in PBS

^d^ heat inactivation (56°C 30 min)

^#^ significant difference to wt in active sera (*p* < 0.001)

§ significant difference to Δ in active sera (*p* < 0.001
