# Supplementary figures and images for: The immunoglobulin M-degrading enzyme of Streptococcus suis, IdeSsuis, is involved in complement evasion
Source: Vet Res. 2015 Apr 19;46(1):45. doi: 10.1186/s13567-015-0171-6 (PMC4404118; doi:10.1186/s13567-015-0171-6)

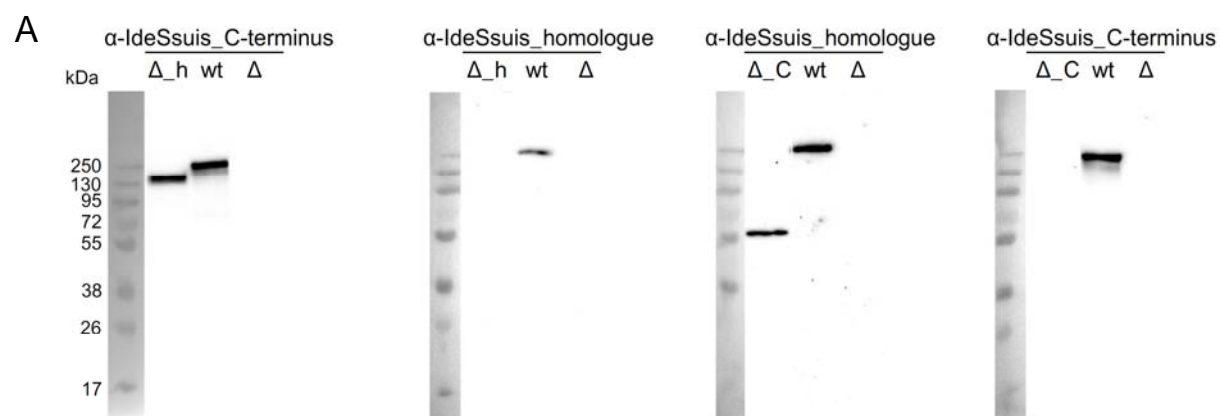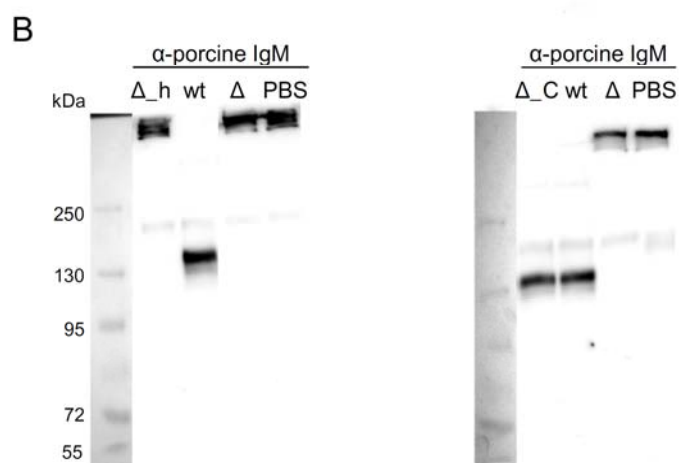

Supplement: Additional file 4: — 10Δide Ssuis _ homologue (Δ_h) and 10Δide Ssuis _ C-terminus (Δ_C) release stabile fragments of Ide Ssuis into the supernatant in accordance with IgM cleavage activity in the case of Δ_C. (A) αIdeSsuis_C-terminus and αIdeSsuis_homologue Western blot analysis of culture supernatants of 10Δide Ssuis _homologue (Δ_h), 10Δide Ssuis_C-terminus (Δ_C), 10Δide Ssuis (Δ) and wild type strain 10 (wt). (B) 10Δide Ssuis_C-terminus but not 10Δide Ssuis _homologue exhibits IgM-cleaving activity. αIgM Western blot analysis of diluted porcine serum incubated with concentrated culture supernatants of 10Δide Ssuis_homologue (Δ _h), 10Δide Ssuis _C-terminus (Δ_C), 10Δide Ssuis (Δ) and wild type strain 10 (wt) or with PBS. [file 13567_2015_171_MOESM4_ESM.pdf]

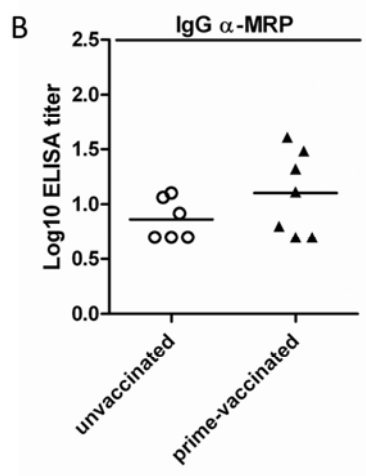

Supplement: Additional file 6: — Immunological analysis of piglets used for the bactericidal assay (see Figure 7 ). (A) α-S. suis serotype 2 (ST2) IgM and (B) α-MRP IgG titers were determined in serum samples of these 7 growing piglets (prime-vaccinated) and for comparison in unvaccinated piglets. Prime vaccination was conducted with a S. suis serotype 2 bacterin. Significant differences are indicated (** p < 0.01). Horizontal lines represent mean values. [file 13567_2015_171_MOESM6_ESM.pdf]
