# Supplementary material for: The immunoglobulin M-degrading enzyme of Streptococcus suis, IdeSsuis, is involved in complement evasion
Source: Vet Res. 2015 Apr 19;46(1):45. doi: 10.1186/s13567-015-0171-6 (PMC4404118; doi:10.1186/s13567-015-0171-6)
Supplement: Additional file 5: — Immunological analysis of piglets used in the experimental infection (see Figure 6 ). (A) α-S. suis serotype 2 (ST2) IgM, (B) α-ST2 IgG titers and (C) α-MRP IgG titers were determined in serum samples of piglets after prime vaccination with a S. suis ST2 bacterin (before experimental infection) and as a control in unvaccinated piglets. Significant differences are indicated. Mean values are shown by horizontal lines. [file 13567_2015_171_MOESM5_ESM.pdf]

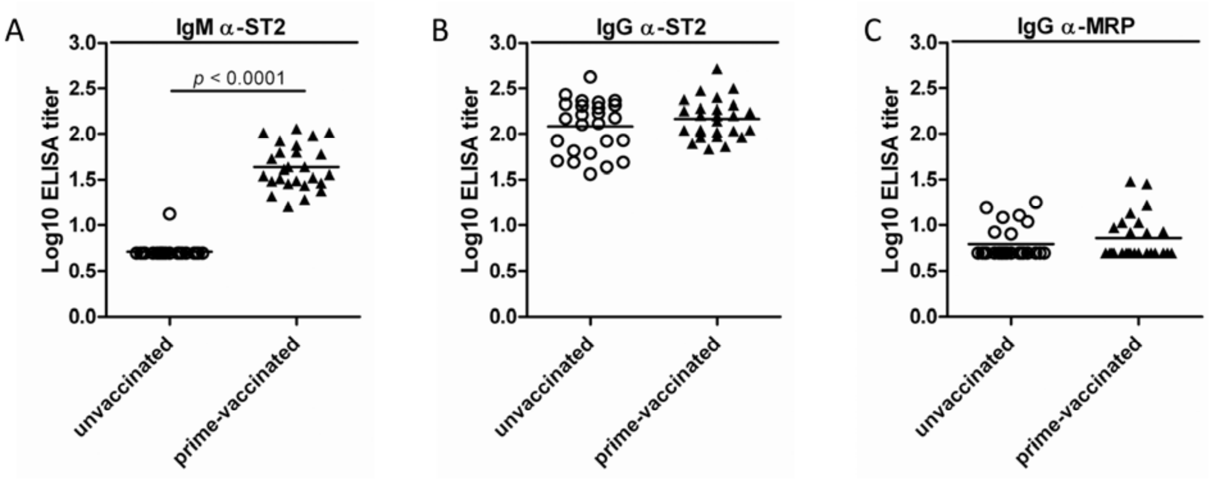

A
IgM α-ST2
p < 0.0001
Log10 ELISA titer
3.0
2.5
2.0
1.5
1.0
0.5
0.0
unvaccinated
prime-vaccinated
B
IgG α-ST2
Log10 ELISA titer
unvaccinated
prime-vaccinated
C
IgG α-MRP
Log10 ELISA titer
unvaccinated
prime-vaccinated
